# Development of the Biological Variation In Experimental Design And Analysis (BioVEDA) assessment

**DOI:** 10.1371/journal.pone.0236098

**Published:** 2020-07-20

**Authors:** Jenna Hicks, Jessica Dewey, Yaniv Brandvain, Anita Schuchardt

**Affiliations:** 1 Department of Biology Teaching and Learning, University of Minnesota, Minneapolis, Minnesota, United States of America; 2 STEM Education Center, University of Minnesota, St. Paul, Minnesota, United States of America; 3 Department of Ecology, Evolution, and Behavior, University of Minnesota, St. Paul, Minnesota, United States of America; 4 Department of Plant and Microbial Biology, University of Minnesota, St. Paul, Minnesota, United States of America; University of the Pacific - Arthur A Dugoni School of Dentistry, UNITED STATES

## Abstract

Variation is an important concept that underlies experimental design and data analysis. Incomplete understanding of variation can preclude students from designing experiments that adequately manage organismal and experimental variation, and from accurately conducting and interpreting statistical analyses of data. Because of the lack of assessment instruments that measure students’ ideas about variation in the context of biological investigations, we developed the Biological Variation in Experimental Design and Analysis (BioVEDA) assessment. Psychometric analyses indicate that BioVEDA assessment scores are reliable/precise. We provide evidence that the BioVEDA instrument can be used to evaluate students’ understanding of biological variation in the context of experimental design and analysis relative to other students and to their prior scores.

## Introduction

Biological systems and the data that is produced from them are inherently variable due to the complexities of these systems and the diverse interactions among entities within the system. Investigators must manage both the variation that is inherent to the system, such as genetic variation between organisms, and variation introduced by the investigative process, such as pipetting error. Experts can implicitly recognize the importance and contribution of variation to topics in biology and the implications for the design of experiments and data analysis. However, students have difficulty integrating the abstract concept of variation into their mental model of biology [1].

To date, the majority of published investigations that characterize students’ understanding of variation from a biological perspective focus on endogenous variation in the context of genetics, evolution, and natural selection [2–7]. Understanding the diverse sources of variation in the context of experimental design and data analysis in biology is an essential skill [8] but is less well-characterized in the extant literature. Here, we describe the development of an assessment tool, hereafter termed the Biological Variation in Experimental Design and Analysis (BioVEDA) assessment. The BioVEDA assessment is intended to be used to evaluate student understanding of variation in the context of biological investigations relative to other students and to prior performance. We present evidence that BioVEDA assessment scores are reliably precise and evidence that the assessment can be used for these two purposes.

### Design of the BioVEDA assessment

The incomplete characterization of students’ conceptual and quantitative understanding of variation necessitates an assessment tool that can be easily used to measure student understanding of variation in experimental design and data analysis [2,7,9]. Assessment tools have been developed to evaluate students’ ability to complete individual phases of a biological investigation, such as the design of experiments or the use of statistics to analyze data [10–16]. None of the available tools ask for the consideration of variation throughout multiple phases of biological investigations. To meet this need, we have developed the Biological Variation in Experimental Design and Analysis (BioVEDA) assessment.

To maximize the ease of use for instructors as well as educational researchers, a multiple-choice format was a priority. We prioritized couching assessment items in a biological context. The rationale for this draws from theory on situated cognition, which emphasizes that knowledge is linked to the context in which it was developed [17]. Therefore, students may not intuitively apply their understanding of statistical concepts (as learned in a statistics course) to analyze and make conclusions about biological data [18].

### Content domain of the BioVEDA assessment: Understanding variation in biological investigations

The BioVEDA assessment is intended to measure one dominant ability: the respondent’s understanding of variation in a biological investigation. This ability domain is necessarily broad, as the consequences of variation are pervasive throughout the investigative process. When conducting biological investigations, variation must be acknowledged, described, and accounted for to allow for valid, replicable, and generalizable conclusions to be made [19]. In this study, variation is conceptualized in a broad sense across both the design and data analysis phases of an investigation.

The importance of the integrated treatment of variation during both the data analysis and design phases of an investigation is exemplified in commonplace practices of biological researchers, such as performing a power analysis to determine the appropriate sample size to detect treatment differences based on anticipated effect sizes. Additionally, statistical results of one experiment or study are often used to inform the design of subsequent investigations, requiring researchers to integrate statistical knowledge with experimental design strategies in different contexts. The omnipresence of myriad sources of variation in biological investigations requires researchers’ attention during both the design and data analysis phases, and coordination of approaches in the two phases is critical so conclusions about the phenomenon under investigation are supported by the data.

To exemplify how an asynchronous consideration of variation between phases could result in inappropriate conclusions being drawn, let us examine an example. Suppose that measurement inaccuracy is a known contributor to experimental variation, so a researcher decides to measure each sample three times each (technical replicates) to get a representative measurement value for the sample. In the data analysis phase, the researcher *should* average these measurements and treat the mean measurement value as one data point in subsequent analyses. If the researcher were to treat each of the three measurements as individual data points, sample size would be inflated, and the variation between replicates that should represent measurement variability would now be inappropriately attributed to variability between individuals.

The BioVEDA assessment probes students’ ability to: 1) acknowledge, identify, and account for variation in the design of an experiment, and as part of data analyses: 2) represent or interpret representations of variability (either graphically or mathematically), and 3) interpret variability in the context of statistical analyses of data [20]. The underlying core concept is students’ understanding of and management of variation. In the sections below, we describe the content domain of the BioVEDA assessment related to each of these three areas. We also detail documented student difficulties related to these topics.

#### Accounting for variation during experimental design

In the design phase of a biological investigation, a researcher identifies sources of variation and attempts to control for sources of unwanted variation. In the laboratory, researchers often try to use model organisms with very similar genetic backgrounds to minimize the influence of genetic and/or phenotypic variation on the experimental outcome. If a researcher aims to leverage the genetic and/or phenotypic variation of a population in their study, they may implement a large sample size to adequately capture this variation. To minimize unwanted environmental variation, one might place control and treatment samples side-by-side in a greenhouse or animal housing facility, with the intent of making environmental conditions (e.g., exposure to sunlight) as similar as possible between the two samples. When experimental variation (sometimes termed measurement error) is a concern, researchers may increase the number of technical replicates performed and average the measurement values or use a more precise instrument to minimize the impact of unwanted experimental variation. The variation in the measured outcome detected in a biological investigation is the cumulative effect of all of these types of variation (i.e. genetic variation, environmental variation, experimental variation) and any additional unexplained variation [19,20]. For each investigation they conduct, biological researchers must consider the sources of variation that are likely to impact the experimental outcome and which sources of variation are important to model or control so the results can be generalizable, yet precise.

Undergraduate students’ understanding of variation in the context of biological experimental design and data analysis is incompletely characterized, despite variation being inherent to biological experimentation. Studies that have investigated students’ ability to design experiments to answer biological questions suggest that students at many levels, from primary to undergraduate, fail to consider variation when designing an experiment [10,21]. Identifying that organisms within a population vary on a genetic or phenotypic level is a reported area of difficulty for students, evidenced by claims that a certain sample of experimental subjects will eliminate naturally occurring variability between subjects [6,10,21]. Additionally, designing experiments that account for sources of variability can be challenging [10,21]. Many students do not consider the amount of phenotypic variation in a population. When dealing with a population with a large amount of phenotypic variation, many students do not design experiments to address this variation by sampling a large number of individuals and by replicating the treatment conditions [10]. Measurement inaccuracy is another potential source of variation in a biological investigation that students can more easily identify [22].

#### Generating and interpreting representations of variation

Variation is equally central to the analysis of data after it has been collected. A first step in data analysis is often the representation of variation in the measured outcome. This may entail visual or graphical representations of data to examine the distribution and spread of the data, or the calculation of summary statistics that mathematically represent variation in the data (e.g. standard deviation, variance, or standard error of the mean) [23,24]. Some graphical representations (e.g. histograms) typically allow for easier visualization of variation in a data set than others (e.g. bar plots). Similarly, different summary statistics provide different information about the variation in a population. For example, the standard deviation provides information about the amount of variation in the measured outcome, whereas the standard error of the mean provides information about the precision of the estimate. An investigator will choose the most appropriate representation of variation for the research question under investigation.

Students who are instructed on algorithmic methods for determining measures of central tendency and amount of variation in the data may prioritize algorithmic ‘plug and chug’ style thinking over the conceptual ideas that summary statistics signify (e.g. the concept of representativeness) [25,26]. When pre-instruction college biology students were asked to construct a graph of biological data to support a claim, many students did not calculate and plot standard summary statistics (e.g. group means) on their graphs and instead chose to plot individual replicate values or the sum of the replicate values [27]. In this case, it would be more appropriate to draw generalizable conclusions from data in aggregate form by calculating and comparing means to account for variation between individual data points. After instruction featuring quantitative thinking, nearly all students constructed graphs that plotted calculated quantities (e.g. the mean) [27]. Though constructing appropriate representations of variation can be challenging for some students, these concepts are not inherently esoteric. Primary school students are able to reason about these summary statistics without explicit statistical instruction on how these values are calculated [9,26].

#### Considering variation in the statistical analysis of biological data

The statistical analysis of data, an essential and informative component of most biological investigations, requires consideration of variation [20]. Statistics are used to measure, model, and control for variation in an investigation to make predictions and explanations about the phenomenon being studied [19,20]. An understanding of the sources of variation present in a biological experiment and the methods that have been implemented to account for said variation is essential so that statistical techniques can be applied appropriately to model and explain the data.

Using statistics to test hypotheses is a nearly ubiquitous practice of biologists (and other scientists), albeit one in which errors are often made. Statistical tests and resulting p-values are often misused and misinterpreted by both students and experts [18,28–32]. The majority of these studies characterizing students’ ideas about the statistical analysis of data have done so in a non-biological context [28,31,33]. Comparatively, fewer studies have described students’ ideas about applying and interpreting statistical tests on biological data. Specific characterization of students’ understanding of statistical analysis of biological data is important because students may not transfer knowledge across disciplinary contexts [34].

The transfer of statistical knowledge to biological contexts requires students to recall mathematical concepts and manipulations and use these ideas in a novel setting to make sense of biological data. This process of transfer requires students to practice interdisciplinary thinking and translate between types of representations (mathematical/numerical to verbal/conceptual) to draw conclusions from data [34,35]. BioVEDA assessment items leverage multiple types of representations (pictorial, mathematical, graphical, and verbal) to query students’ understanding of variation as it relates to the statistical interpretation of data.

### Study objective

The objective of this study is to develop a test that assesses students’ understanding of variation in the context of biological investigations. Here, we describe the development of an assessment that fulfils the above criteria. We present evidence that BioVEDA assessment scores are reliable/precise, and consistent across semesters of administration. We also present evidence that students’ assessment scores can be used to determine their degree of understanding on the above concepts and to measure changes in student learning after instruction on these topics.

## Methods

### Participants

All students that were interviewed or assessed for the purposes of this study were enrolled in either a lecture or laboratory introductory biology course at a large, Ph.D. granting Midwestern university. Students typically enroll in these courses during their freshman or sophomore year and may enroll in the laboratory and lecture courses concurrently or separately. The introductory biology laboratory curriculum is presented in a two-course sequence. The first semester course features scripted inquiry experiments and focuses on experimental design, data analysis, and technical skills. The second semester course is a Course-Based Undergraduate Research Experience (CURE), where students design and carry out an independent biological investigation. We provide demographic information about the students and teaching assistants at this institution so instructors and researchers in other contexts may use this information to draw their own conclusions about the generalizability of our findings to their contexts. The student pool that is enrolled in these courses is 65% female and 35% male, 20% domestic students of color, and approximately 20% first-generation college students. Graduate teaching assistants (TAs) for the course were also included in the study. The graduate student pool from which TAs are hired is 55% female, 16% students of color, and 26% international students. This study is approved under IRB#: STUDY00003137.

In all semesters, students and TAs were recruited to take the assessment and participate in this study via their laboratory course instructor, who provided students with a link to take the assessment electronically via Qualtrics. Students enrolled in the first semester course completed the assessment during class time on the first day of class (referred to as the pre-first-semester student responses). Students enrolled in the second semester course completed the assessment outside of class at the end of the semester (these are referred to as the post-second-semester student responses). TAs completed the assessment before starting their teaching appointment. Data was collected over the course of 5 semesters (Spring 2018 –Spring 2020). At the time of taking the assessment, students and TAs had the option to decline to have their assessment data used in the study. Approximately 90% of students and TAs consented to have their data included in the study. Students were incentivized to complete the assessment by the awarding of course points that constituted less than .5% of their course grade. Students could earn points for completion of the assessment without consenting to participate in the study.

### Design and development of the BioVEDA assessment

We established a list of specific content goals that were at the intersection of variation, biological experimental design, and data analysis by identifying core ideas specified in the literature on these topics (see Introduction). Faculty that teach undergraduate introductory biology, introductory biology laboratory, and biostatistics courses were consulted to validate that these topics should be prioritized and included on the BioVEDA assessment tool. Additionally, as part of another study, interviews were conducted with undergraduate students enrolled in the second semester introductory biology laboratory course. Students were interviewed about sources, explanations, and impacts of variability on their study. Common areas of difficulty were compiled, cross-referenced with the list of core ideas generated from reading the literature, and used as an additional means by which to prioritize certain core ideas for inclusion on the BioVEDA assessment. A summary of the topics that are targeted in the BioVEDA assessment is included in Table 1. Some items incorporate multiple topics and are included in multiple topic categories. Underlying all of these topics is an understanding of variation (Table 1). Multiple-choice questions were drafted using best practice guidelines to target these prioritized core ideas [36,37]. The drafted questions were then used in small-scale and large-scale administrations in introductory biology laboratory courses to assess the difficulty of items and the quality of distractor options (described below).

**Table 1:** Summary of topics targeted by the BioVEDA assessment.

Investigative Phase	Topic	Related Literature	Related Questions	Average Proportion Correct ^a^
Experimental Design	Identifying sources of variation in an experiment (e.g. genetic, environmental, measurement, etc.)	[10,38]	1, 7	0.66
	Controlling for different sources of variation in an experiment (e.g. genetic, environmental, measurement, etc.)	[10,21,22]	2, 3, 4, 6	0.74
	Understanding the relationship between sample size and genetic variation in a biological data set	[39–41]	5	0.75
Data Analysis	Representing observed variation in a data set	[9,18,42]	13, 14	0.61
	Understanding how observed variation impacts the outcome of statistical tests	[28]	8, 9, 10, 11	0.45
	Interpreting p-values generated by statistical tests	[18,31–33]	12, 14, 15, 16	0.51

^a^ Average proportion of correct responses are calculated using responses from post-second-semester biology laboratory students.

#### Small-scale pilot administration to assess quality of distractors

The initial draft of the BioVEDA assessment was administered to post-second-semester students in Spring 2018. Student responses (*N* = 152) were analyzed to determine the proportion of students who correctly answered each item and very difficult items (fewer than 10% of students answered correctly) were discarded. Other items were eliminated on the basis of the quality of distractor options, such that all of the remaining questions had distractor options that were chosen by at least 5% of students. Items that we wished to include on the next version of the BioVEDA assessment that did not perform well (e.g. items that targeted prioritized concepts but were written in such a way that certain distractor answers were not chosen by any students) were revised.

#### Large-scale assessment administration to assess item difficulty and quality of distractors

Sequential versions of the BioVEDA assessment items that remained after expert review, the student think-aloud interviews, and the pilot study item analysis were administered to pre-first-semester students in the Fall 2018 (*N*_*FA18*_ = 138) and Spring 2019 (*N*_*SP19*_ = 292). Student responses on the assessment were examined to determine 1) the distribution of student responses amongst the answer options, 2) the proportion of students who answered the item correctly, and 3) how well the item distinguished between the top 25% and the bottom 25% of students [43,44]. Items that did not perform well (e.g., had distractor options that were not selected, were answered incorrectly by more than 90% of students, or did not discriminate well between high- and low-performing students) were dropped or revised for subsequent administrations. Two items underwent revision after the Fall 2018 semester, and one of those items underwent an additional revision after the Spring 2019 semester. The final version of the BioVEDA assessment contains 16 items. The assessment questions are included in S1 File.

### Summary of analyses to provide evidence of validity

The BioVEDA assessment is intended to be used to evaluate the degree of students’ understanding of variation relative to other students, or to examine changes in students’ understanding of variation after instruction. Table 2 presents a summary of procedures implemented to support the assertion that the BioVEDA assessment can be used for these intended purposes [45]. Below, we address multiple sources of validity evidence.

**Table 2:** Summary of approaches to provide evidence of validity.

Type of Validity Evidence	Question	Methodology that addresses
Evidence based on test content	Does the assessment appropriately represent the specified knowledge domain?	Expert review of items, literature review, Rasch analysis
Evidence based on response processes	Are the thinking processes thought to be used to answer the items the ones that were actually used?	Think-aloud student interviews
Evidence based on internal structure	Do the items capture the intended number of dimensions or constructs?	Confirmatory factor analysis
Evidence based on relations to other variables	Are scores on the assessment predictive of some external criterion measure?	Known-groups comparison between graduate TAs and undergraduate students

Table structure and content modified from [45,46].

#### Expert review

To provide evidence that the BioVEDA assessment appropriately represents the specified knowledge domain, the BioVEDA questions were submitted to four faculty members for expert review (one with a Ph.D. in learning sciences and a Ph.D. in biology, one with a Ph.D. in biology with expertise in biostatistics, and two with Ph.Ds. in biology with expertise in teaching science process skills in biology CURE laboratories). These faculty evaluated the questions on the basis of 1) the importance of the concept being assessed, 2) the scientific and statistical accuracy of the question stem and answer options, and 3) the clarity of the phrasing in the question stem and answer options. Questions were revised for accuracy and clarity following expert review and used in subsequent assessment administrations.

#### Student think-aloud interviews

Student think-aloud interviews were used to determine that students are correctly interpreting the BioVEDA questions and selecting their answers based on the intended thought processes (as opposed to answering based on unintended non-content clues such as choice of phrasing). Think-aloud interviews were conducted with twenty students from a first-semester introductory biology lecture course in the Fall 2018 semester. Students who are enrolled in this course are typically in their freshman or sophomore year and are at a similar level to the students in the first-semester laboratory course. Students were selected on a volunteer basis, and they did not receive compensation for their participation. Each student was asked to complete five of the multiple-choice questions and to describe their thought process and reasoning out loud. Each question was answered by at least 5 students. Students were prompted to explain why the answer they selected was the best option, and why the other options were not optimal. Students were asked if there were any phrases or features of the included diagrams or graphs that were unfamiliar or unclear. Any phrases that were not familiar or unclear to multiple students were revised before the next stage of validation. At least 80% of the students interpreted the question stem and answer options as the authors intended for all of questions used for interviews. One area of difficulty that was reported and subsequently revised was the inclusion of the mathematical expression for a t-statistic. The initial version included standard mathematical symbols that some students reported to be unfamiliar (e.g. $\bar{x}$ to indicate the sample mean), and the revised version included words or phrases instead of symbolic representations (e.g. *mean* instead of $\bar{x}$).

#### Confirmatory factor analysis

The BioVEDA assessment was designed to measure a single dominant ability: students’ understanding of variation as it applies to a biological investigation. To investigate the dimensional nature of the BioVEDA assessment, we assessed the fit of confirmatory factor analysis models to the data. This analysis was conducted on post-second-semester students’ dichotomously scored data collected during the Spring 2019 and Fall 2019 semesters (*N*_*SP19*_ = 109, *N*_*FA19*_ = 254, *N*_*Total*_ = 354). Given the low number of factors being tested (one or two) and the number of items loading on each factor (16 for the one-factor model, 7 and 9 for the two-factor model), the sample size has been generally recognized by others to be sufficient to perform factor analysis [47]. Post-second-semester student responses were used for this analysis because students’ answer selections were presumed to be less influenced by guessing than on the pre-first-semester administration (when students are less likely to have an accurate understanding of the concepts assessed by the BioVEDA test). Models were fit using the ‘lavaan’ package version 0.6–5 in R version 3.6.3 [48,49]. Weighted Least Squares (WLS) was used to estimate the model parameters. Latent factors were standardized, allowing for free estimation of all factor loadings. Model fit was assessed using three model-fit statistics (the Comparative Fit Index (CFI), Tucker-Lewis Index (TLI), and the Root Mean Square Error of Approximation (RMSEA)). We compared the fit of a unidimensional model and a two-dimensional model using a Chi-square test with the null hypothesis that the models fit equally well, and the outcome of the Chi-square test was used to select the best fitting model.

#### Item response theory analysis

Item Response Theory (IRT) was used to examine the degree to which the BioVEDA assessment appropriately measures the knowledge domain of ‘variation in biological investigations’ that we aim to measure. A Rasch analysis was performed on the post-second-semester student dichotomously scored responses (*N* = 354) to determine person ability and item difficulty scores. Models fitted to the data under IRT assume that test performance is due to person abilities and various characteristics of the individual item, including how difficult the item is, how well an item discriminates between respondents of high and low ability, and the extent to which responses are due to guessing [43,50–52]. Rasch models comprise a category of IRT models that assume the observed performance on an assessment is a product of person ability and only one item characteristic: the difficulty of the items. The person ability score takes into account a respondent’s performance on items of different difficulty, and all items are not presumed to be equal in difficulty (as is true when comparing raw percentage or sum scores from a multiple-choice assessment).

A unidimensional Rasch model was fitted to the data using the ‘mirt’ package version 1.32.1 in R version 3.6.3 [48,53]. Model fit was assessed by examining item and person infit and outfit statistics. The Rasch model was then used to compute ability scores for each respondent and difficulty estimates for each item to determine if the items can adequately capture the ability range of the respondent population being measured.

#### Assessing the suitability of using raw BioVEDA scores to indicate ability

We recognize that researchers and instructors may be interested in using the BioVEDA assessment to evaluate students’ understanding of variation in biological investigations but may not have the time or the ability to conduct a Rasch analysis to generate person ability measures. To determine whether percentage scores could be used as an alternative to Rasch ability measures, a Pearson’s correlation was computed between respondents’ percent correct scores and their person ability scores calculated using the Rasch model. High correlation between the two values would indicate that the percentage scores could be used to report on students’ performance on the assessment [13,54].

#### Known-groups comparison

A known-groups comparison was used to determine whether groups that should logically differ in their performance do indeed show differential performance on the assessment [55]. Graduate TAs (*N*_*TA*_ = 32) have more experience and instruction on experimental design and statistical analysis of data than the undergraduate students they teach, so they can be expected to perform better on the assessment. Similarly, students who have completed two semesters of biology laboratory courses (*N*_*Sem2*_ *=* 354, data from Spring 2019 and Fall 2019) featuring instruction on variation in biological investigations can be expected to perform better than students who are just beginning their first semester of a biology laboratory course (*N*_*Sem1*_ *=* 384, data from Fall 2019 and Spring 2020). Therefore, BioVEDA assessment scores were compared across these three groups of respondents (TAs, post-second-semester students, and pre-first-semester students). Samples of students were selected such that each assessment response was from one unique student, satisfying the assumption of sample independence. Raw percentage scores were compared across groups using a one-way ANOVA. Post-hoc comparisons of individual groups were done using Tukey’s HSD. We used an alpha level of .05 for all statistical tests.

#### Analysis of learning gains

The BioVEDA assessment is intended to be used to detect changes in learning over time, perhaps in response to instruction. The curriculum for both the first- and second-semester lab courses contain several laboratory exercises that require students to consider and account for variation. Participation in these courses is thus expected to elevate students’ understanding of variation in biological investigations. A paired sample t-test on students’ (*N* = 282) pre-first-semester and post-second-semester assessment scores was used to determine whether the BioVEDA assessment can be used to detect learning gains (e.g. those assumed to occur after participating in this two-semester lab course series).

### Summary of analyses to provide evidence of reliability/precision

Below, we describe the analyses performed to provide evidence that BioVEDA assessment scores are reliable/precise. We use Item Response Theory to describe the reliability/precision of the assessment. We provide evidence of internal consistency and evidence of consistency across administrations to different groups of students.

#### Rasch test information, standard error, and reliability

One advantage of using Item Response Theory (e.g. the Rasch model described above) is that reliability is not measured as a uniform index for all possible respondents. Instead, reliability/precision is measured as a function of person ability, with the assumption that measurement precision is not constant across all ability levels. To describe the reliability/precision of BioVEDA assessment scores, we calculate the test information function and conditional standard error of measurement function using the Rasch model specified above. The Rasch model can also be used to calculate a Rasch reliability index (also termed the Person reliability). The Rasch person separation reliability index can be interpreted similarly to the Cronbach’s alpha or KR-20 indices of internal consistency: values range from 0–1, with higher values indicating higher internal consistency [56,57]. The Rasch person reliability index for the BioVEDA assessment is .68, which is reasonable for an assessment measuring a broad conceptual domain [57,58].

#### Cronbach’s alpha and item-total correlations

Cronbach’s alpha is commonly used, often in incorrect ways, as a measure of assessment reliability [58]. Cronbach’s alpha is best interpreted as a measure of internal consistency; a measure of the degree to which all items on an assessment elicit a similar response pattern [58,59]. However, the BioVEDA assessment is intended to test a range of knowledge related to variation as it applies to experimental design. This is a broad topic and by necessity covers a broad range of experimental design and data analysis practices (Table 2). Therefore, such assessments would not be expected to yield very high alpha values which might be expected of attitudinal constructs [58]. Despite these considerations, the Cronbach’s alpha for the post-second-semester student responses on the BioVEDA assessment is .69 (95% CI = .64, .74) which has been reported as falling in the moderate to acceptable range and is on par with other multiple-choice concept assessments [58].

Item-whole correlations are reported in S2 File. Both the raw correlation (correlation of the focal item with the entire scale, including itself), and the correlation with the remaining items in the scale (excluding the focal item) are reported.

#### Consistency across semesters

The BioVEDA assessment should enable instructors and researchers to consistently measure students’ understanding regardless of when the test is given. Test/retest strategies are often used to demonstrate reliability of test scores over time. However, this method cannot be employed in the context of this study as students begin receiving instruction on the topics assessed by the BioVEDA test immediately after the semester begins. The instruction students receive is assumed to affect their understanding of variation in biological investigations, which is assumed to affect their score on the BioVEDA assessment. Therefore, we implemented an alternative strategy to investigate the stability of BioVEDA assessment scores across administrations. The proportion of students who answer each item correctly was compared between the Spring 2019 and Fall 2019 second-semester course administrations by computing a Pearson’s correlation. The mean ability score of students in each semester was also compared using a two-tailed t-test.

## Results

### BioVEDA assessment scores can be used to measure introductory students’ understanding of variation in biological investigations

Multiple approaches were used to provide evidence that the BioVEDA assessment can be used to measure students’ understanding of variation in a biological investigation. A summary of these approaches is presented in Table 2, and the sections below describe sources of validity evidence.

#### The BioVEDA assessment appears unidimensional

The BioVEDA assessment was designed to measure one dominant ability: students’ understanding of variation in a biological investigation. Confirmatory factor analysis models were fitted to the data to explore the dimensional structure of the assessment. The unidimensional model (including the final versions of the 16 multiple-choice questions) specified that all questions load on a single factor, hereafter termed ‘Understanding of Variation.’ The model fit is acceptable, with a CFI of .93, a TLI of .92, and an RMSEA of .04 (90% CI = .02, .05). All indicators have significant loadings, and the standardized factor loadings are positive, ranging from .21 to .80 (Table 3).

**Table 3:** Factor loadings for the unidimensional confirmatory factor analysis.

Item	*β*	SE	*p*-value
1	0.58	0.07	***
2	0.47	0.07	***
3	0.73	0.05	***
4	0.31	0.08	***
5	0.21	0.08	**
6	0.80	0.05	***
7	0.50	0.07	***
8	0.50	0.07	***
9	0.30	0.08	***
10	0.22	0.08	**
11	0.55	0.07	***
12	0.55	0.07	***
13	0.35	0.07	***
14	0.59	0.06	***
15	0.33	0.07	***
16	0.40	0.07	***

*Abbreviations*: *β* = Standardized factor loading, SE = standard error for *β*

** = *p* < 0.01

*** = *p* < 0.001.

Although the BioVEDA assessment is designed to measure one dominant ability, students’ understanding of variation in a biological investigation can be organized into two subdomains: variation in the design phase and variation in the data analysis phase. The assessment is designed to include questions that interrogate respondents’ understanding of variation in both of these phases of a biological investigation (Table 1). Therefore, it is plausible that the assessment could be measuring two dimensions, namely, ‘Design’ and ‘Data Analysis.’ Items 1 through 7 relate to experimental design, and items 8 through 16 relate to data analysis, so a two-dimensional model was specified to reflect this structure. The fit statistics for the two-dimensional model are similar to those of the unidimensional model (CFI = .93, TLI = .92, RMSEA = .04 (90% CI = .02, .05). A scaled Chi-squared difference test indicates that the models fit the data similarly well (χ^2^(1) = 2.6, *p* = .11). Since the model fit indices are similar between the unidimensional and two-dimensional models, we default to the more parsimonious (unidimensional) model to describe the structure of the assessment. These results support the theoretical design of the assessment and indicate that the 16 items measure one underlying dominant ability that represents ‘understanding of variation in experimental design and data analysis.’

#### The BioVEDA assessment adequately measures student ability

The results of the confirmatory factor analysis indicate that the 16 BioVEDA items collectively measure a single dominant ability or dimension, so a unidimensional Rasch model was fitted to the data (*N* = 354 post-second-semester student responses) to estimate item difficulty and person ability level. Item difficulty was estimated for each item (Table 4 shows difficulty estimates and standard error). Model fit was assessed by examining item mean-square infit and outfit statistics (Table 4), and person mean-square infit and outfit statistics. Mean-square infit/outfit values less than 0.5 indicate model overfit and those greater than 1.5 indicate model underfit; ideal values are close to 1.0 [60]. The average mean-square infit is 0.93, and the average mean-square outfit is 0.89. All of the 16 items meet criteria for acceptable fit [60], and therefore do not misfit the model. The average person mean-square infit is 0.91 (with 98% of respondents falling in the acceptable range), and the average person mean-square outfit is 0.89 (with 92% of respondents falling in the acceptable range) [60]. No respondents had infit or outfit statistics larger than 2.0 (above which may distort or degrade the model) [60].

**Table 4:** Item difficulty and fit statistics for the Rasch model.

Item	Difficulty	SE	Item Infit	Item Outfit
1	-0.05	0.12	0.85	0.85
2	-0.45	0.13	0.92	0.90
3	-1.22	0.14	0.86	0.75
4	-1.56	0.15	1.01	0.99
5	-1.29	0.14	1.05	1.05
6	-1.70	0.15	0.85	0.66
7	-1.62	0.15	0.95	0.87
8	-0.66	0.13	0.91	0.87
9	0.95	0.13	0.95	1.00
10	0.25	0.12	1.00	1.00
11	0.38	0.13	0.87	0.85
12	-0.08	0.12	0.88	0.87
13	-0.42	0.13	0.97	0.94
14	-0.59	0.13	0.86	0.82
15	0.08	0.12	0.96	0.95
17	0.37	0.13	0.94	0.93

*Abbreviations*: SE = standard error for the estimate of item difficulty, Item Infit = mean-square infit, Item Outfit = mean-square outfit.

**Fig 1:**
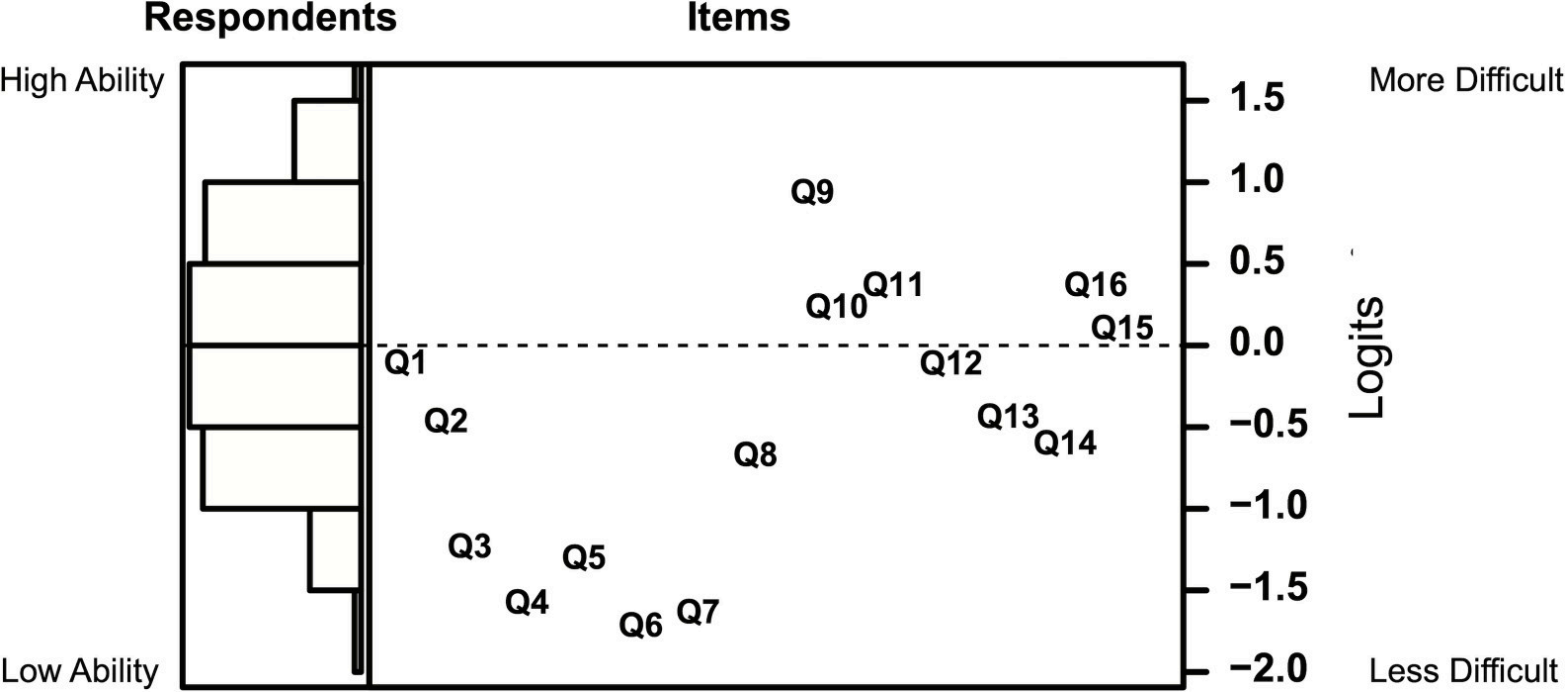
Wright map of respondent ability and item difficulty calculated using the Rasch model. *N* = 354 respondents. Respondents are plotted on the left in a binned histogram, and items are plotted on the right. The axis for both respondent ability and item difficulty (in logits) is noted on the right. Dashed line indicates the mean respondent ability.

Ability estimates for all respondents were computed using the Rasch model, and displayed beside the item difficulty values in a Wright map [57] (Fig 1). The mean score (for both person ability and item difficulty) is scaled to 0 and indicated by a dashed line. Respondents are displayed on the left, and items are plotted on the right. Items that are above the dashed line are more difficult than the average item, and items below the line are less difficult than average. Similarly, respondents with higher than average ability are above the dashed line, while respondents with lower than average ability are below the dashed line. Respondents and items are plotted on the same scale such that the position of an item on the scale indicates that a person of that ability has a 50% chance of answering that item correctly. A person of average ability (ability = 0) has a greater than 50% chance of answering items below the dashed line correctly, and a less than 50% chance of answering items above the dashed line correctly.

Person ability and item difficulty measures calculated using the Rasch model can be compared to evaluate the test targeting of the assessment. A well-targeted assessment will have items that range in difficulty such that they can adequately capture the ability of the intended respondent population. A rule of thumb for comparing the mean item difficulty (*M* = -0.48) and the mean person ability (*M* = 0.00) suggests there should be less than a 1.00 logit difference between the mean item difficulty and mean person ability, which is the case for this data set [61]. Rasch measures can also be used to examine potential floor or ceiling effects by assessing the percentage of respondents at the highest or lowest measure of the instrument [62]. There are no respondents in this sample that score below the lowest measure of the instrument (minimum item difficulty = -1.70), suggesting that there is no floor effect with this assessment in this sample of respondents. A small proportion of respondents (9%, *N* = 32) score above the highest measure of the instrument (maximum item difficulty = 0.95), indicating that there may be a ceiling effect for this sample of respondents.

#### Raw BioVEDA scores are a reasonable indicator of person ability

A Pearson’s correlation was computed between the respondents’ percent correct score and the person ability score calculated using the Rasch model. The correlation is very high (*r* = .99, *p* < .001), indicating that the raw percentage scores can provide a satisfactory indicator of students’ understanding of variation in a biological investigation.

#### The BioVEDA assessment can distinguish between naïve and advanced respondents

**Fig 2:**
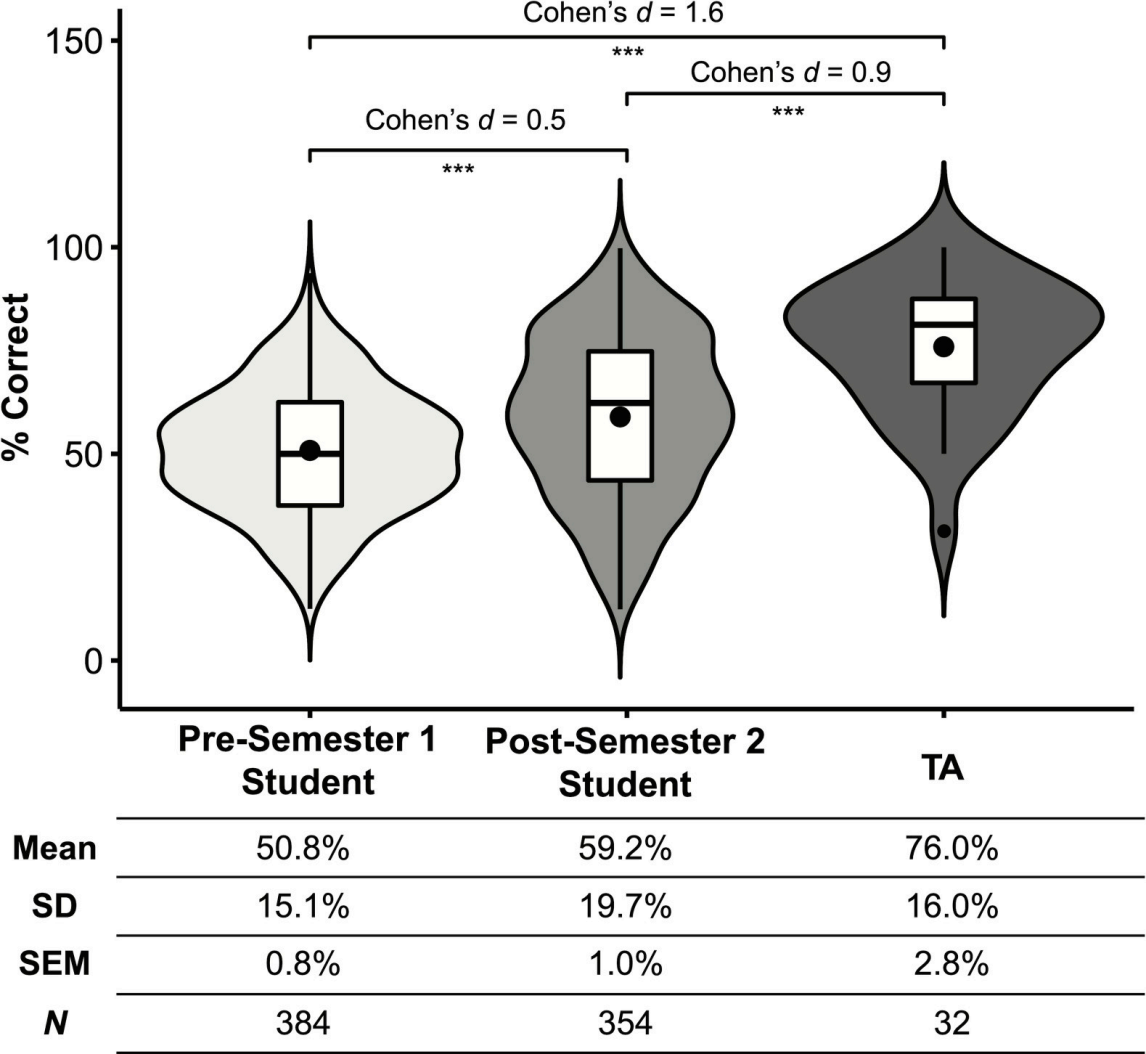
The BioVEDA assessment can distinguish between groups with different ability levels. Violin plots with inlaid box plots of BioVEDA scores (% correct). Pre-first-semester students are shown in light gray, post-second-semester students are shown in medium gray, and TAs are shown in dark gray Horizontal bar in the middle of the box indicates the median score for each group. Large black dot in center of boxplot indicates the mean score for each group. *** = *p* < .0001. Effect size shown as Cohen’s *d*. The table below the graph shows summary statistics of each group’s assessment performance (SD = Standard Deviation, SEM = Standard Error of the Mean).

The BioVEDA assessment is intended to be used to detect differences in content knowledge between individuals. To investigate the extent to which BioVEDA assessment scores can discriminate between respondents who should vary in their ability, BioVEDA scores were compared between first-semester students (pre-instruction), second-semester students (post-instruction), and graduate teaching assistants that served as the primary instructors for the first-semester lab course. Compared to undergraduate students, graduate students are assumed to have more advanced understanding of variation as it relates to a biological investigation, since they have completed more coursework in biology and have direct experience with conducting biological investigations. Similarly, undergraduate students who have completed two semesters of introductory biology lab courses are expected to have more advanced understanding of variation in biological investigations than students who have not yet completed any semesters of biology lab courses. As expected, BioVEDA scores differ significantly across the three groups of respondents (*M*_*Sem1*_ = 51%, *M*_*Sem2*_ = 59%, *M*_*TA*_ = 76%; one-way ANOVA, *F*(2, 767) = 43.72, *p* < .0001) (Fig 2). Post-hoc comparisons via Tukey’s HSD indicate that the differences between each group are also significant (Fig 2). The magnitude of the difference between first- and second-semester students (Cohen’s *d* = 0.5) is smaller than the difference between second-semester students and graduate TAs (Cohen’s *d* = 0.9). This aligns with the differences in experience and knowledge that one would expect for these groups. These data indicate that the BioVEDA assessment can be used to detect differences in respondents’ understanding of variation.

We envision that the BioVEDA assessment can be used to measure individual changes in learning over time. To determine if BioVEDA assessment scores can detect changes in learning that occur after instruction on the topics assessed in the test, we examined students’ pre-first-semester and post-second-semester scores using a paired sample t-test (*N* = 282). We detect a significant increase in students’ BioVEDA scores after two semesters of instruction (*M*_*PreSem1*_ *=* 51%, *M*_*PostSem2*_ = 61%; *t*(281) = 8.88, *p* < .0001). The difference between the pre-first-semester and post-second-semester scores represents a medium sized effect (Cohen’s *d* = 0.53). These data indicate that BioVEDA scores can be used to detect individual changes in learning when administered as a pre- and post- test.

### BioVEDA scores are reliable/precise

Multiple approaches were used to provide evidence that the BioVEDA assessment provides reliable/precise measurements of students’ understanding of variation in a biological investigation. The sections below describe evidence that BioVEDA scores are reliable/precise and consistent.

#### Reliability/Precision from the Rasch model

**Fig 3:**
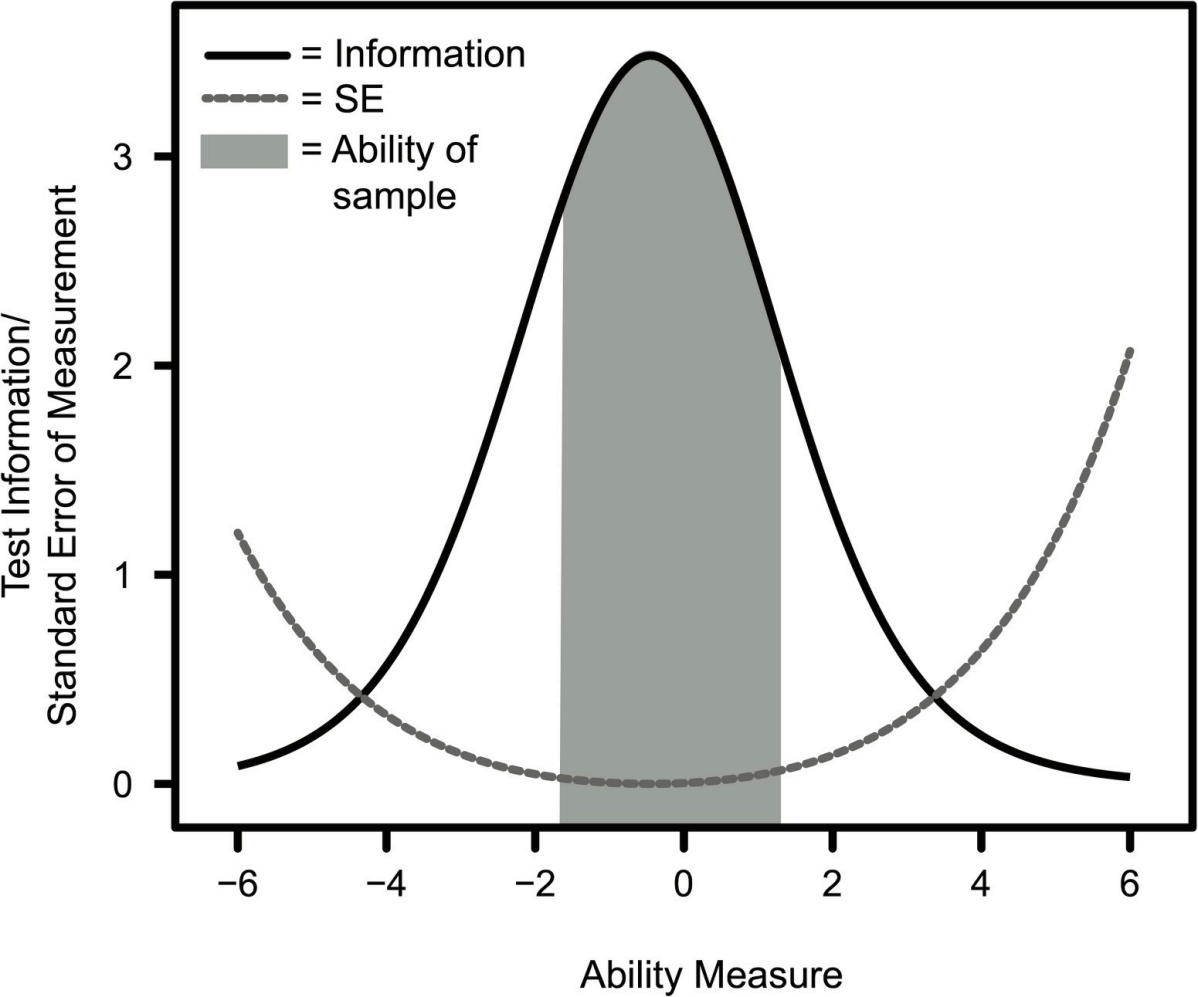
Test information and standard error calculated using the Rasch model. The black solid line indicates test information for persons at the indicated ability measure, and the gray dashed line indicates the standard error of the estimate for persons at the indicated ability measure. The gray shaded area under the information curve indicates the ability range of respondents in our sample.

Item Response Theory models (e.g. Rasch models) can be used to describe the reliability/precision of measurement of a test. Measurement precision/reliability that is determined using Item Response Theory is expressed as a function of ability. It is not assumed that the test will be equally reliable/precise for persons at different ability measures. Test precision can be interpreted by examining both the test information function (Fig 3, solid black line) and the standard error of measurement function (Fig 3, dashed gray line). Test measurements are most precise/reliable when test information is high and standard error of measurement is low. The BioVEDA assessment provides the most information for people of near-average ability levels (ability of approximately 0) (Fig 3). The standard error of the estimate is lowest for individuals of average ability, indicating that estimates of these persons’ ability level are the most precise (Fig 3). The BioVEDA assessment provides less precise information for people of very high or very low ability measures.

#### The BioVEDA assessment is consistent across semesters

In order for an assessment to be used to measure student content knowledge in different contexts, the assessment must produce consistent results. We evaluated the consistency of BioVEDA scores across test administrations at the question and whole-assessment level. Student performance on the BioVEDA items was compared between the Spring 2019 and Fall 2019 post-second-semester test administrations. All test administrations were done in the same laboratory course, and students from different semesters have similar preparation and thus ability on the BioVEDA assessment. Indeed, the correlation between the proportion of correct responses on each item in each semester is high (Pearson’s *r* = 0.98, *p* < .0001). This indicates that the BioVEDA assessment produces consistent results across semesters. Additionally, comparing the mean percent correct score across semesters, there were no significant differences across undergraduate student total scores (*M*_*SP19*_ = 61%, *SEM*_*SP19*_ = 2%, *M*_*FA19*_ = 58%, *SEM*_*FA19*_ = 1%; two-tailed t-test, *t*(201) = 0.94, *p* = .34, Cohen’s *d =* 0.11), which provides additional evidence that BioVEDA assessment scores are consistent.

## Discussion

The purpose of this study was to develop an assessment that could be used to measure students’ relative degree of understanding of variation as it pertains to a biological investigation. We have developed the BioVEDA assessment; a 16-item test that fulfils that objective. The BioVEDA assessment was built on a literature review of student difficulties with variation in the context of biological or scientific investigations. This study provides multiple sources of evidence of validity (Table 2) [46]. Experts in biostatistics, learning sciences and biology reviewed the instrument, providing evidence that the assessment appropriately represents the specified knowledge domain (understanding of variation in biological investigations). Additionally, the instrument was examined by think-aloud interviews with students to establish that students are interpreting the questions as intended and are using appropriate and relevant thought processes to select their answers. Response data were analyzed using confirmatory factor analysis, and the results support a single underlying dominant ability. This is consistent with the intended design of the assessment, that the items measure a unidimensional construct, namely students’ understanding of variation in experimental design and data analysis Acceptable item and person infit and outfit statistics from the Rasch model suggested that there was good model–data fit.

We provide evidence that the assessment can be used to evaluate students’ understanding of variation relative to other students, and to their prior performance. A known-groups comparison between pre-first-semester undergraduate students, post-second-semester undergraduate students, and graduate teaching assistants indicates that the BioVEDA assessment is able to detect predicted differences in understanding of the content (Fig 2). In addition, there are statistically significant differences in students’ pre-first-semester and post-second-semester BioVEDA scores, providing evidence that BioVEDA scores can be used to measure changes in student learning.

The BioVEDA items span a broad content domain (Table 1). This is by necessity, as variation must be considered at many points during a biological investigation. The broad nature of the content domain is reflected by the measures of internal consistency provided here (Rasch person reliability index, Cronbach’s alpha, and item-total correlations). Though some of the corrected (if-dropped) item-total correlations are low, these items query topics that are essential to encapsulating the content domain: identifying environmental variation, describing the utility of a large sample size with respect to the amount of variation observed, and understanding the impact of variation (as measured by standard error) in a statistical test. Loosely associated items are commonly accepted for tests that measure a broad conceptual domain [58].

The proportion of students who answer each item correctly is consistent across semesters. Additionally, students’ scores are similar across semesters, indicating that the BioVEDA is consistent over time at the whole-assessment level. Taken together, these data indicate that the BioVEDA assessment scores are consistent, and can be used to measure students’ relative degree of understanding of variation in biological experimental design and analysis.

At this time, the BioVEDA assessment has not been examined to determine the degree to which the measurements of the knowledge domain with this instrument align with measurements of the knowledge domain generated using different tools or assessments. Existing assessment tools do not measure students’ understanding of variation in the same way that the BioVEDA assessment does (asking students to apply both their understanding of variation to the design and the analysis phases of a biological investigation), making it difficult to appropriately align BioVEDA scores with external measurements of a similar knowledge domain. As more assessment tools become available in the future, it may be feasible to collect this type of validity evidence.

### Intended uses for the BioVEDA assessment and study limitations

BioVEDA assessment scores are intended to be used as a holistic indicator of introductory biology students’ understanding of variation in a biological investigation. Scores may be compared in aggregate across courses or groups of students to examine differences in their degree of understanding (as in Fig 2), or as a pre- and post- test to measure learning gains in response to instruction (as reported here). Both of these intended uses are relatively low-stakes testing scenarios. We do not intend for BioVEDA scores to be used in high-stakes testing scenarios (e.g. for placement in different levels of coursework, or as a sole indicator for assigning grades). We also do not intend for BioVEDA scores to be used as a criterion-referenced assessment and have deliberately avoided providing cut scores to indicate proficiency levels.

We envision that instructors may desire to use the BioVEDA assessment to identify particular topics of difficulty for students who are in their classes. However, we note that the validation evidence described in this study has been entirely at the whole-test level, and that the topic listings provided in Table 2 do not indicate that these are subscales that can be used for measurement or assessment purposes. Further, the information that can be obtained about students’ understanding of specific topics is bounded by the answer options provided in the multiple-choice question [63]. Individual items which produce interesting response patterns may indicate topics that merit in-depth exploration, perhaps using student interviews or constructed response questions to further probe and characterize students’ ideas and suggest pathways for instructional interventions to enhance student cognition.

We found a very strong correlation between students’ raw percentage scores and the person ability score calculated with the Rasch model, which suggests that instructors may use the raw percentage scores as a representation of students’ ability if conducting a Rasch analysis is not feasible. Instructors or researchers who are interested in using the BioVEDA assessment can access the questions and instructions for use in the S1 File.

The BioVEDA assessment items were developed with the intent of measuring students of a wide range of abilities. Our data indicate that the BioVEDA assessment can discriminate between students of different ability levels in the first- and second-semester introductory laboratory course sequence at this institution. Estimates of respondents’ ability levels are most precise for persons of average or slightly less than average ability and are least precise for persons of high ability (Fig 3). The Rasch analysis suggests that there may be a ceiling effect for our sample of post-second semester students, indicating that the assessment would benefit from the addition of more difficult items. The individual items are distributed over a range of difficulties (least difficult = -1.70, most difficult = 0.95), but the items are not evenly distributed across this range (Fig 1). The BioVEDA assessment will continue to be updated, with particular effort made to generate items of difficulty levels that complement the existing items. Developing more items that evenly cover a greater range of difficulty will allow for more precise ability estimations of a wider pool of respondents. Additionally, questions are not evenly distributed across topic categories (Table 2). Future BioVEDA assessment development will also focus on developing more items to target topic areas which are underrepresented by the current 16 items.

The validity evidence collected in this study was based on responses from students enrolled in an introductory biology laboratory course and the course TAs at one specific institution. In this sample, approximately half of the students had completed a prior statistics course, which may not be representative of introductory biology students at other institutions. Similar approaches to those described here should be undertaken to determine if BioVEDA scores can be used to evaluate other populations of students.

### Conclusions

The BioVEDA assessment scores are reliable and can be used to characterize and evaluate students’ ideas about variation in biological investigations. This instrument is unique in that it asks students to consider variation at multiple parts of the investigative process; how variation can be accounted for in the experimental design and data collection phases as well as how variation impacts the data analysis phase. Researchers or instructors who are interested in using the BioVEDA assessment can access the questions in S1 File.

## Supporting information

S1 File BioVEDA assessment questions with key. (DOCX)

S2 File Item-whole correlations. The ‘Raw Correlation’ column shows the correlation of each item with the total score. The ‘If-Dropped Correlation’ column shows the item-whole correlation for this item against the scale without this item.
(DOCX)

S3 File Manuscript data. (XLSX)
