# Supplementary material for: Development of the Biological Variation In Experimental Design And Analysis (BioVEDA) assessment
Source: PLoS One. 2020 Jul 20;15(7):e0236098. doi: 10.1371/journal.pone.0236098 (PMC7371189; doi:10.1371/journal.pone.0236098)
Supplement: S1 File — (DOCX) [file pone.0236098.s001.docx]

**S1 Supporting Information. BioVEDA assessment questions with key.**

**Instructions for BioVEDA Assessment Administration**

Instructors or researchers may use the BioVEDA assessment questions to characterize students’ ideas about variation in biological experimental design and data analysis. Best choice answers are indicated by bold text below. Users of the BioVEDA assessment are encouraged to administer the assessment in a way that minimizes students’ access to the questions (e.g. via an online survey platform such as Qualtrics) to avoid the correct answers becoming easily searchable. The authors will provide a BioVEDA assessment Qualtrics file to interested users upon request. We ask that users who intend to publish data from administration of the BioVEDA assessment cite this paper.

**BioVEDA Assessment Questions**

Q1

A researcher is interested in the effects of a drug that speeds up cell turnover on the rate of zebrafish fin regeneration. They grow two groups of 20 fish in two different fish tanks. One tank has the drug added to it, and the other does not. The researchers make identical wounds in each fish tail fin, and measure the degree of wound healing in each fish after 1 month. Which of the following is a source of organismal variation in this experiment?

1. The rates of wound healing between the two treatment groups
2. The size of the initial wound in the tail fin between the two groups
3. **The rates of wound healing within the same treatment group**
4. The size of the initial wound in the tail fin within the same treatment group

Q2

Two different groups are measuring the distance between the eyes of zebrafish after removing them from the tank and taking images of them.

Group 1 is using a computer to measure the distance, which is very accurate.

Group 2 is measuring the distance with a ruler marked in mm, which is not very accurate.

Each group gets 30 fish. The class wants to find the best estimate of the population's mean distance between the eyes. Students have two strategy options to accomplish this goal in one class period. What would you advise each group, and why?

Option A: Measure all 30 fish once

Option B: Measure 10 fish, 3 times each

1. Both groups should choose Option B, because it is good scientific practice to do repeated measures of an individual in an experiment.
2. Both groups should choose option A, because it is good scientific practice to sample as many individuals as possible.
3. **Group 1 should choose option A, Group 2 should choose option B, based on the accuracy of the measurement method.**
4. It doesn't matter which method each group chooses, as long as they are consistent throughout their experiment.

Q3

Jane is measuring zebrafish heart rate in response to caffeine treatment. She makes sure that the fish tanks being used for the experiment are the same size, have the same volume of water in them, and are being kept at the same temperature. Why does Jane ensure that the tank setup is similar between the caffeine and comparison groups?

1. **She can be more certain that any effect she sees is due to the caffeine treatment, and not difference in housing conditions.**
2. Keeping the environment constant will minimize the amount of variation between individuals in the same treatment group.
3. It is good scientific practice to ensure that there is no difference in the environment between experiment and comparison groups.
4. She will be able to eliminate variation between the individuals within the caffeine-treated and comparison groups.

Q4


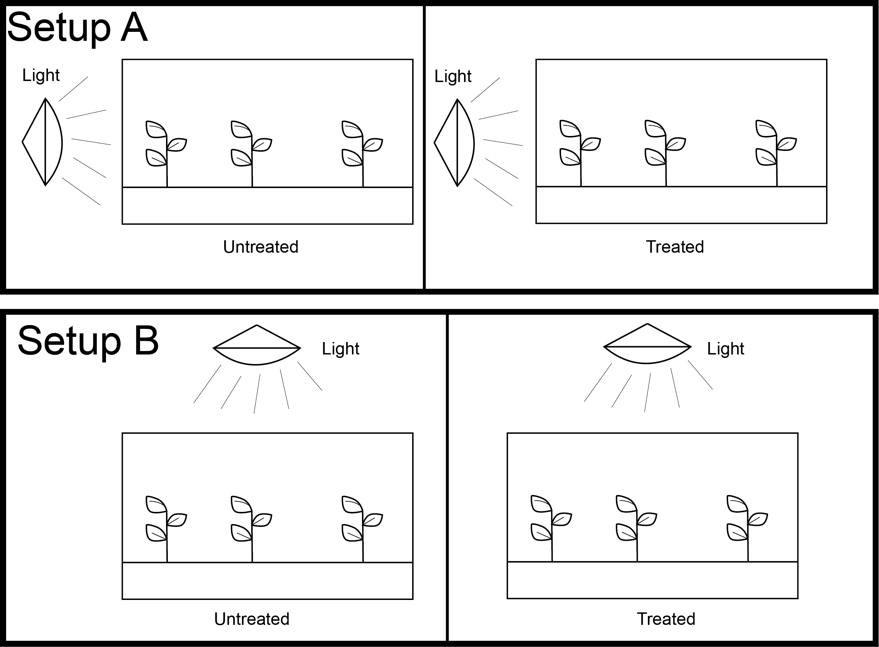
​Sam is testing the effect of a pesticide on plant growth. He grows plants in a terrarium and treats one with pesticide, and one without. He positions a light source next to the terrariums, and waits to see the results. Which light setup (shown in the pictures below) is best?

1. Setup A, because it accurately models natural variation in light intensity within a population.
2. **Setup B, because it limits variation between plants within the untreated and treated groups.**
3. Setup A or B, because the treated and untreated groups have been set up identically.
4. There is not enough information to determine which setup is best.

Q5

A large sample size is desirable because a larger sample size will:

1. Increase the amount of variation between individuals in the sample.
2. **Yield a more accurate estimate of the population being sampled.**
3. Decrease the amount of variation between individuals in the sample.
4. None of the options are true.

Q6 ​

The image below is a representation of the zebrafish population you have access to for your experiment. What would be the best way to sample this population for your experiment?

|  |  |  |  |  |  |  |  | Air Conditioner | |
| --- | --- | --- | --- | --- | --- | --- | --- | --- | --- |
|  |  |  |  |  |  |  |  |  |  |
|  | Rack A | |  | Rack B | |  | Rack C | |  |
|  |  |  |  |  |  |  |  |  |  |
|  |  |  |  |  |  |  |  |  |  |
|  |  |  |  |  |  |  |  |  |  |
|  |  |  |  |  |  |  |  |  |  |
|  |  |  |  |  |  |  |  |  |  |
|  |  |  |  |  |  |  |  |  |  |
|  |  |  |  | Sink Area | | |  |  |  |
| Door | |  |  |  |  |  |  |  |  |

1. **Take fish from all racks equally for both the comparison and experimental groups to account for differences between the racks.**
2. Take only the comparison fish equally from all racks. It does not matter where you get the experimental fish.
3. Take fish from one rack for your control group and another rack for your experimental group to account for difference between the racks.
4. The way you take fish from the racks does not matter because they are all in the same environment already.

Q7


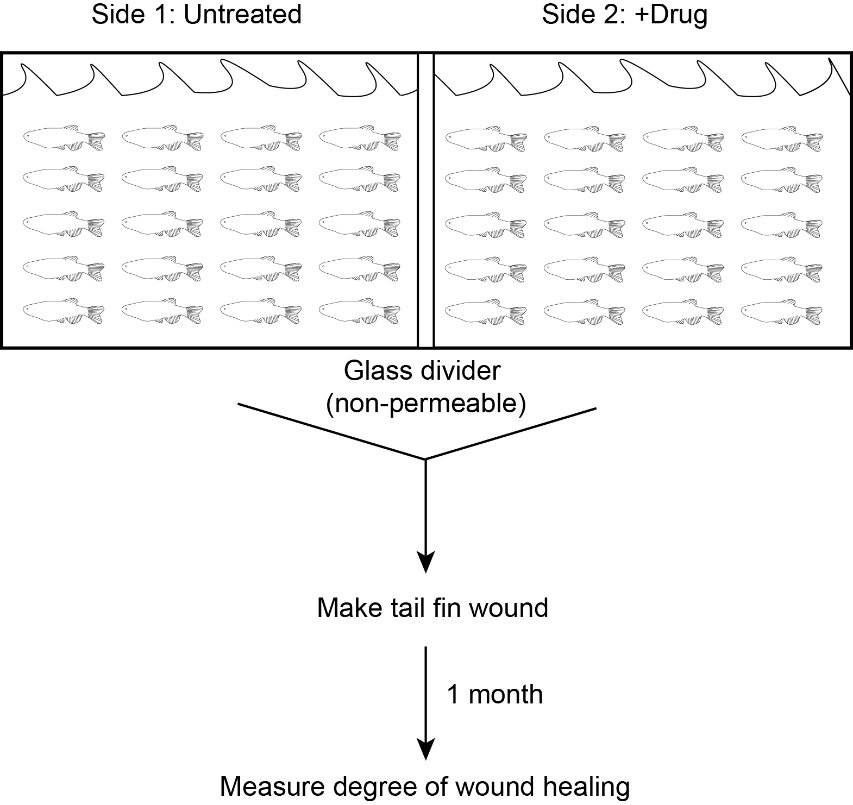
A researcher is interested in the effects of a drug that speeds up the rate of zebrafish fin regeneration. They grow two groups of 20 fish in one fish tank, split in two by a non-permeable glass divider. One side has the drug added to it, and the other does not. The researchers make identical wounds in each fish tail fin, and measure the degree of wound healing in each fish after 1 month.

When the researchers pull the fish out of the tank at the end of a month, they gather data on the wound size for each fish 3 separate times. Repeated data collection will allow the researchers to control for:

1. Variation between organisms.
2. **Variation in measurement accuracy.**
3. Variation in the environment.
4. This control is not necessary.

Q8

The equation below represents how the t-statistic for a two-sample t-test is calculated. Use this equation to answer the following question.


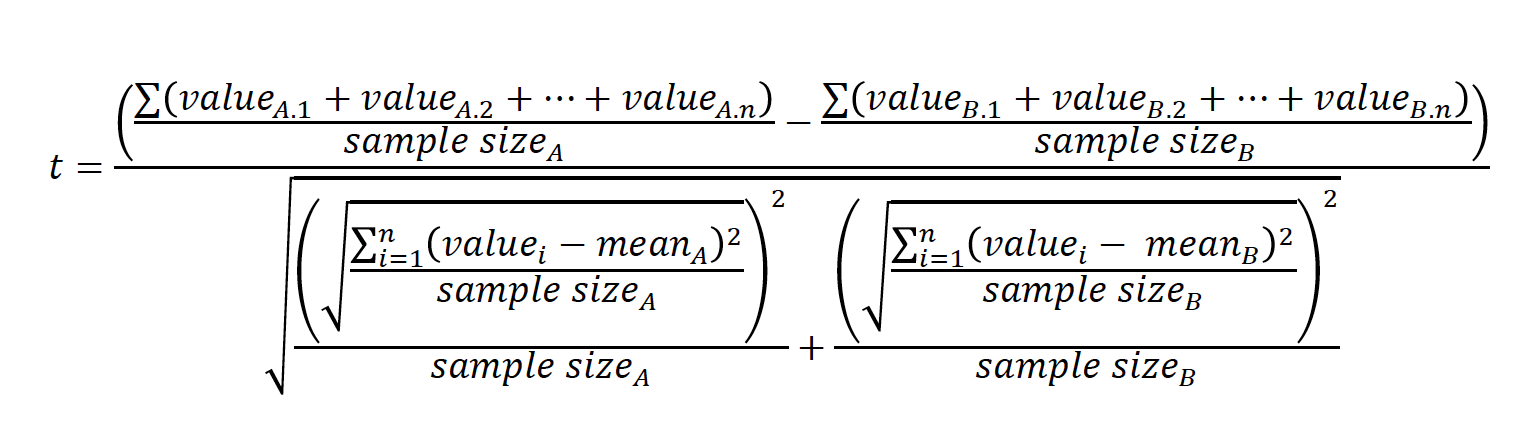


The boxes below are a pictorial representation of values you collected for an experiment. These boxes are to scale. Based on this information, which p-value would you most likely match your data?

Difference between means

Standard Error

1. **This data would result in a small p-value that is likely to be significant.**
2. This data would result in a small p-value that is not likely to be significant.
3. This data would result in a larger p-value that is likely to be significant.
4. This data would result in a larger p-value that is not likely to be significant.

Q9

The equation below represents how the t-statistic for a two-sample t-test is calculated. Use this equation to answer the following question.

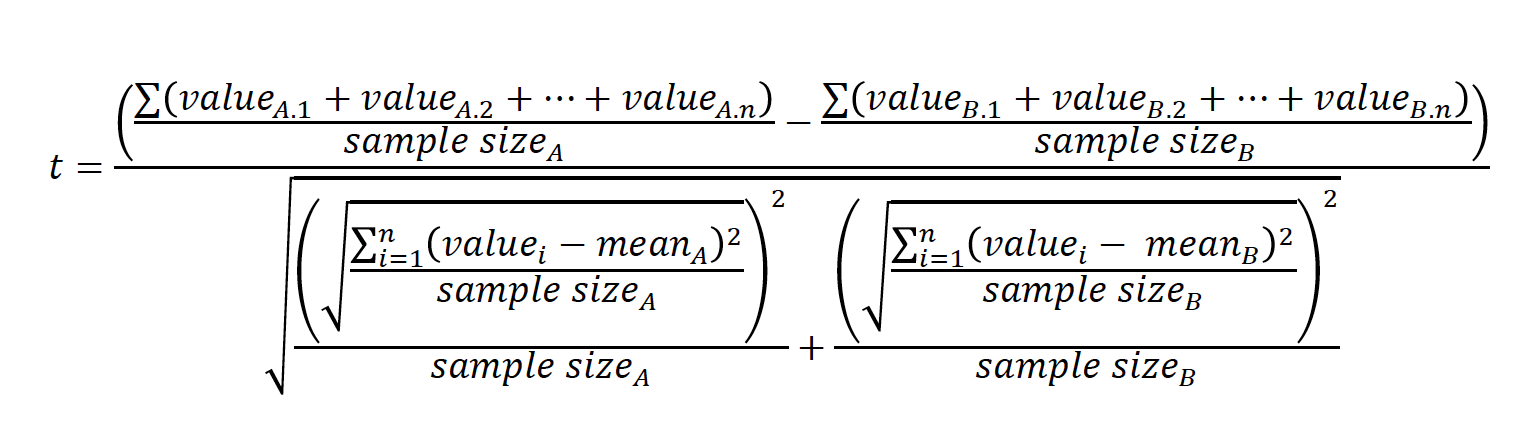

 You perform an experiment testing the effect of caffeine on zebrafish heart rate, with a sample size of 100 fish for both your comparison and experimental groups. Based on the data you collect, you calculate the following values. Based on the values below, what would likely be the correct interpretation of your data?

| **Difference between means** | **Standard Error** |
| --- | --- |
| 10 | 173 |

1. **The t-statistic will be small and the data will likely not be significant.**
2. The t-statistic will be small and the data will likely be significant.
3. The t-statistic will be large and the data will likely not be significant.
4. The t-statistic will be large and the data will likely be significant.

Q10

The equation below represents how the t-statistic for a two-sample t-test is calculated. Use this equation to answer the following question.

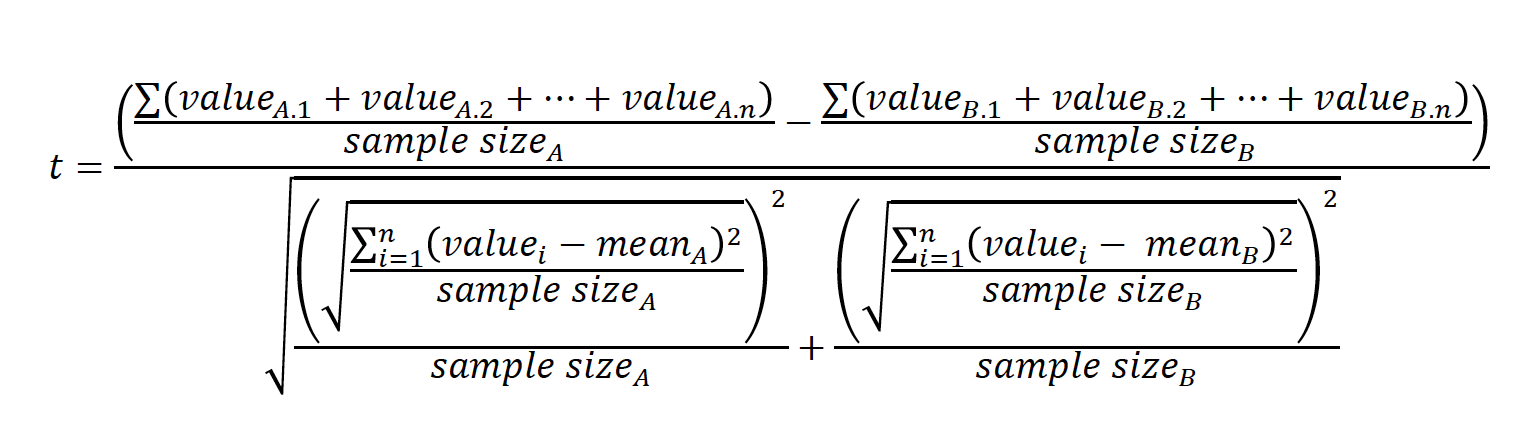
You run an experiment and calculate a t-statistic of 10 for your data. Which of the choices below would likely be the best pictorial representation of your t-statistic? The boxes are to scale.

Difference between means

Standard Error

Standard Error

Difference between means

Difference between means

Standard Error

1. There is not enough information to answer the question

Q11

A researcher is measuring the difference in number of stripes between female and male zebrafish. They analyze 1000 male and 1000 female fish, and find that male fish have 5 stripes, and female fish have 6 stripes. In this population, you do not see any variation in stripe number within each sex. Do you need to perform a statistical test?

1. Yes, because they are comparing two conditions (males vs. females).
2. Yes, because it is good scientific practice to perform statistical tests after data collection.
3. No, because the number of individuals sampled is high (n=1000).
4. **No, because there is no variation in these populations with respect to number of stripes.**

Q12

Your class breaks into 20 different groups that each run a replicate of an experiment testing the effect of an antibiotic of the growth of a bacteria. Each group calculates a p-value, shown in the table below.  Only group 15, bolded in the table, calculates a p-value that is below 0.05.  Group 15 says that the class should conclude that the antibiotic has a significant effect on growth because they found a significant p-value.  Do you agree?

| p-values calculated | | | | |
| --- | --- | --- | --- | --- |
| 0.6 | 0.56 | 0.8 | 0.3 | 0.09 |
| 0.25 | 0.66 | 0.57 | 0.17 | 0.16 |
| 0.34 | 0.7 | 0.35 | 0.61 | **0.02** |
| 0.51 | 0.4 | 0.39 | 0.28 | 0.24 |

1. Yes, any indication of statistical significance should be trusted because it doesn't matter how infrequently you see it.
2. Yes, because the field of science believes it is important to trust any sign of statistical significance in data collected.
3. No, because if you averaged the p-values together, overall the data does not turn out to be statistically significant.
4. **No, because when you run 20 replicates of the same experiment, you'll likely find one significant result by chance.**

Q13


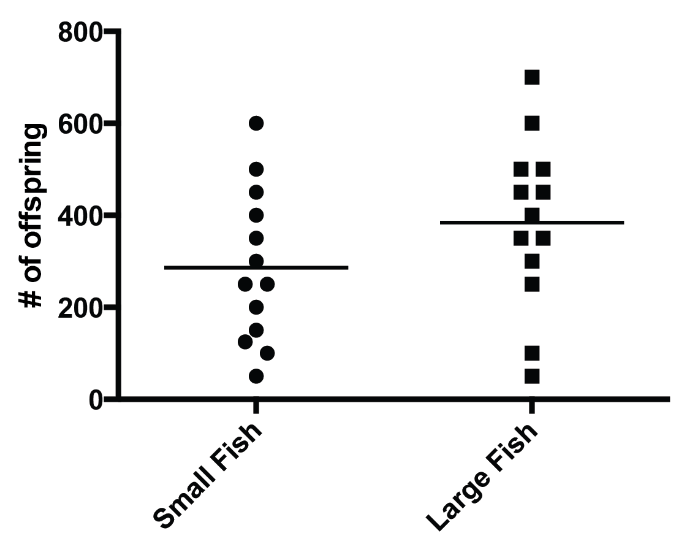
A group of students is testing whether large zebrafish have more offspring than small zebrafish. They collect data on the number of eggs laid by large fish and small fish. Their data is shown below (each point represents one fish, horizontal lines indicate the mean for each group).

 Student A thinks that there will not be any significant difference between the number of offspring for small and large fish. Student B thinks there will be a significant difference. Who do you agree with?

1. Student A, because the means are close to each other.
2. **Student A, because there is a lot of variation in both samples.**
3. Student B, because the means are different from each other.
4. Student B, because there is less variation in small fish.

Q14

Which of the choices below best describes this mathematical expression?

$$\frac{\sum\left| sample value-mean \right|}{sample size}$$

1. The absolute distance between the sample mean and the population mean.
2. The overall absolute spread of the data collected.
3. The average absolute value of the sample data.
4. **The average absolute deviation from the mean of the sample data.**

Q15


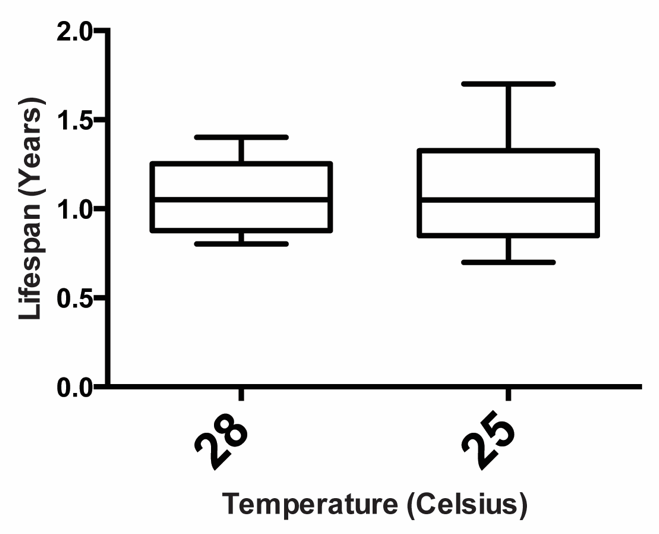
Fred is doing an experiment to test if water temperature affects zebrafish lifespan. He grows 2 tanks of zebrafish, one at 28ºC, and one at 25ºC. He analyzes ***3*** fish per condition. His data is shown below:

Fred performs a t-test to determine whether there is a significant difference between the two treatments, and calculates a p-value of 0.8. What can Fred confidently conclude about his data?

1. **This experiment is inconclusive, because his sample size is too small to yield informative results.**
2. This experiment is inconclusive, because the p-value is greater than 0.05, so his data is not informative.
3. Water temperature doesn't affect lifespan, because there is no significant difference in lifespan of fish grown at the two temperatures.
4. Water temperature does affect lifespan, because the range of lifespan is larger in fish grown at 25ºC.

Q16


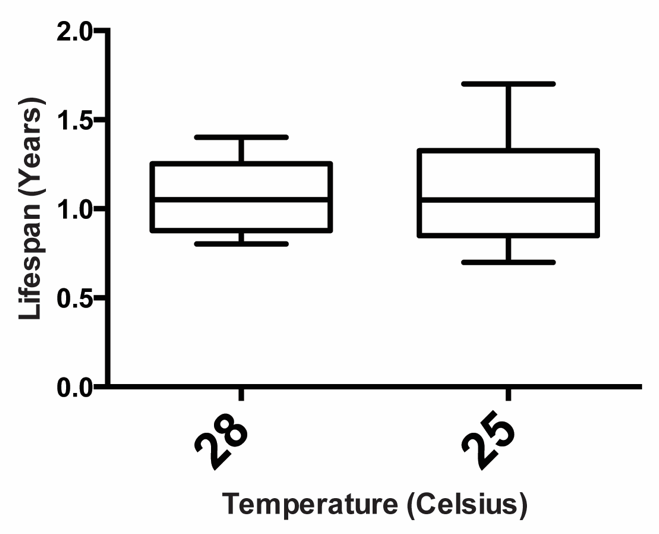
Fred is doing an experiment to test if water temperature affects zebrafish lifespan. He grows 2 tanks of zebrafish, one at 28ºC, and one at 25ºC. He analyzes ***300*** fish per condition. His data is shown below:

Fred performs a t-test to determine whether there is a significant difference between the two treatments, and calculates a p-value of 0.8. What can Fred confidently conclude about his data?

1. This experiment is inconclusive, because the p-value is greater than 0.05, so his data is not informative.
2. This experiment is inconclusive, because his sample size is too small to yield informative results.
3. Water temperature does affect lifespan, because the range of lifespan is larger in fish grown at 25ºC.
4. **Water temperature doesn't affect lifespan, because there is no significant difference in lifespan of fish grown at the two temperatures.**
