# Supplementary material for: Development of the Biological Variation In Experimental Design And Analysis (BioVEDA) assessment
Source: PLoS One. 2020 Jul 20;15(7):e0236098. doi: 10.1371/journal.pone.0236098 (PMC7371189; doi:10.1371/journal.pone.0236098)
Supplement: S2 File — The ‘Raw Correlation’ column shows the correlation of each item with the total score. The ‘If-Dropped Correlation’ column shows the item-whole correlation for this item against the scale without this item. (DOCX) [file pone.0236098.s002.docx]

**S2 Table.** **Item-whole correlations.** The ‘Raw Correlation’ column shows the correlation of each item with the total score. The ‘If-Dropped Correlation’ column shows the item-whole correlation for this item against the scale without this item.

| **Item** | **Raw Correlation** | **If-Dropped Correlation** |
| --- | --- | --- |
| 1 | 0.52 | 0.39 |
| 2 | 0.43 | 0.30 |
| 3 | 0.53 | 0.42 |
| 4 | 0.30 | 0.18 |
| 5 | 0.25 | 0.12 |
| 6 | 0.55 | 0.45 |
| 7 | 0.39 | 0.27 |
| 8 | 0.45 | 0.32 |
| 9 | 0.35 | 0.22 |
| 10 | 0.32 | 0.16 |
| 11 | 0.49 | 0.36 |
| 12 | 0.49 | 0.35 |
| 13 | 0.37 | 0.22 |
| 14 | 0.52 | 0.40 |
| 15 | 0.38 | 0.23 |
| 16 | 0.40 | 0.26 |
